# Logical Fallacies in Missed Diagnosis of Stridorous Patients: A Case Report

**DOI:** 10.7759/cureus.62456

**Published:** 2024-06-15

**Authors:** Everett Young, WayAnne Watson, Priya Krishna

**Affiliations:** 1 Otolaryngology, Loma Linda University School of Medicine, Loma Linda, USA; 2 Otolaryngology, Loma Linda University Medical Center, Loma Linda, USA

**Keywords:** dyspnea, generalized anxiety, paradoxical vocal fold motion, tracheal stenosis, laryngomalacia

## Abstract

Tracheal stenosis and paradoxical vocal fold motion are both common laryngological diagnoses that can present with similar symptoms of dyspnea. Co-morbid psychiatric issues can complicate diagnostic accuracy and lead to logical fallacies in the attribution of symptom etiology. We present a case of a 38-year-old female who presented repeatedly to the emergency department with respiratory distress, inspiratory stridor, wheezing, and anxiety. On examination, she had stridor that appeared to correlate with episodes of elevated anxiety and bedside laryngoscopy which showed intermittent paradoxical vocal fold motion. A computed tomography scan showed 40% narrowing of the distal tracheal lumen, but symptoms were felt to be inconsistent and out of proportion to stenosis. She was seen several more times in the ED and eventually followed up in the laryngology clinic, where she had a tracheoscopy showing Cotton Meyer grade III stenosis. This unique case highlights the logical fallacies that may lead to misdiagnosis when evaluating stridorous patients with comorbid personality and anxiety disorders.

## Introduction

Tracheal stenosis occurs due to the narrowing of the tracheal lumen and is most commonly caused by prolonged endotracheal intubation or tracheostomy. Tracheal stenosis is diagnosed in patients with symptoms of dyspnea, wheezing, and stridor with flexible bronchoscopy showing narrowing of the trachea [1]. Acquired laryngomalacia is a condition rarely found in adults that is characterized by a collapse of supraglottic structures during inspiration. Diagnosis is made with dynamic laryngoscopy in patients with stridor and dyspnea on exertion [2]. Both conditions may be difficult to differentiate from other conditions that share similar symptoms. One such condition is paradoxical vocal fold motion (PVFM), also referred to as vocal cord dysfunction, which presents with similar clinical manifestations such as wheezing, stridor, and dyspnea [3]. PVFM occurs due to the closure of the vocal cords during inspiration and is commonly triggered or exacerbated by psychological stress or disorders, which makes the diagnostic process even more difficult in patients presenting with comorbid anxiety that can exacerbate dyspnea [4]. PVFM is diagnosed with laryngoscopy that shows adduction of the vocal folds during inspiration and the formation of a small posterior diamond-shaped glottal chink [3]. We present the case of a patient with dyspnea whose symptoms were initially attributed to PVFM and anxiety, subsequently diagnosed with tracheal stenosis. We discuss the diagnostic challenge of parsing out the medical and psychiatric components of patients presenting with symptoms of dyspnea and anxiety or other psychiatric complaints.

## Case presentation

**Figure 1:**
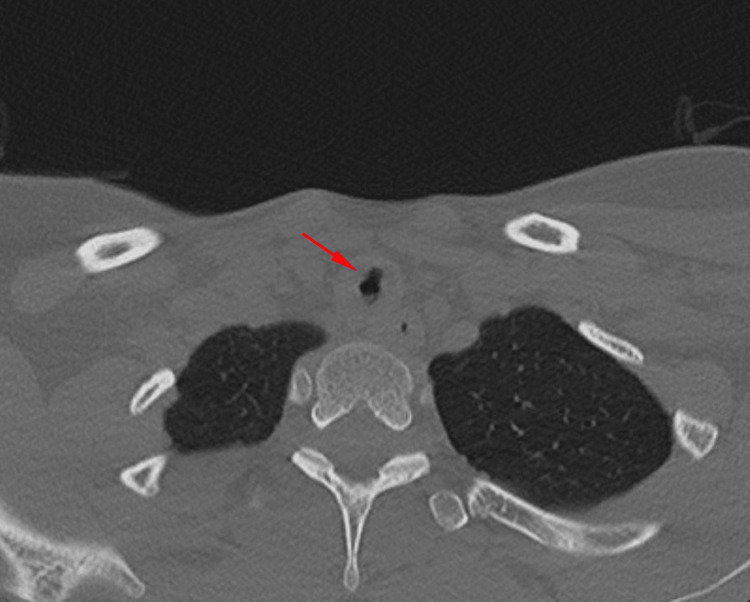
CT soft tissue neck showing 40% tracheal narrowing 6.5 cm below the laryngeal inlet

A 38-year-old female presented to the Emergency Department (ED) with progressively worsening shortness of breath and wheezing for the past six weeks. The patient had been intubated four times in the past year for seizures and respiratory distress relating to alcohol and benzodiazepine overdose and/or withdrawal, with the most recent intubation occurring one month prior to presentation. The patient had a medical history significant for anxiety, depression, bipolar disorder, as well as benzodiazepine and alcohol use disorder. At the time of examination, the patient was not forthcoming with her complete medical history and the reasons for her repeated intubations. On physical exam, the patient exhibited respiratory distress, tachypnea, wheezing, and inspiratory stridor. She had no oxygen desaturations on room air and other physical examination findings and laboratory results were unremarkable. The patient repeatedly asked nursing and physician staff for benzodiazepines and IV opioid medications while in the ED. Flexible laryngoscopy showed paradoxical motion of the vocal folds during inspiration, consistent with PVFM. A CT soft tissue scan of the neck with contrast showed 40% short segment narrowing of the trachea, approximately 6.5 cm below the laryngeal inlet (Figure 1). The on call ENT attending and senior resident reviewed the CT scan and exam findings and diagnosed the patient with PVFM disorder, recommending SLP and laryngology follow-up. Though narrowing was noted on the CT scan, her symptoms were thought to be out of proportion to the narrowing. Bronchoscopy was not performed at the initial visit. The patient was discharged but presented again to the ED three more times over the following week. Given the patient’s previous psychiatric history, psychiatry was consulted, and the patient was diagnosed with an unspecified anxiety disorder. She followed up in the laryngology clinic with continued shortness of breath, where tracheoscopy was performed, which showed very distal Cotton-Meyer grade III tracheal stenosis. The patient was then directly admitted for urgent microsuspension direct laryngoscopy (MSDL), balloon dilation, and kenalog injection of the tracheal stenosis. Following the procedure, the patient reported improved breathing. Prior to discharge, she underwent repeat MSDL in the operating room, which showed no significant stenosis or tracheomalacia, and had no further desaturations on continuous pulse oximetry.

The patient's condition slowly worsened over the next few months, with multiple ED visits and frequent contact of the laryngology clinic staff. Several months later, the patient underwent MSDL with balloon dilation of the trachea and kenalog injection due to grade III tracheal stenosis found 5.7 cm distal to the vocal folds. Two months later, the patient underwent outpatient bronchoscopy, which showed mild mid-tracheal cicatricial stenosis and laryngomalacia with mild arytenoid edema. Following the procedure, the patient became obtunded, unresponsive, and tachycardic with concern for seizures. She was intubated and admitted to the Intensive Care Unit (ICU), then extubated the following day and discharged two days later. The patient presented again three days later after an alcohol overdose and requested a tracheostomy, which was performed by the ENT service. She left the ICU against medical advice three days later but presented one month later after her tracheostomy tube spontaneously fell out. The tube was replaced, but the patient presented again ten days later due to hemoptysis and continued shortness of breath. No evidence of prior hemoptysis was noted on exam, but laryngoscopy was notable for arytenoid prolapse over the glottic opening and redundant supraglottic mucosa. The patient continued to request specific IV opioid medications and IV benzodiazepines. Due to her continued shortness of breath and exam findings of laryngomalacia, laser supraglottoplasty with removal of redundant arytenoid mucosa was performed. The patient has had subsequent improvement in her breathing symptoms, though at this point she was exhibiting actual findings of PVFM and underwent Botox injection of the true vocal folds, with minimal improvement.

## Discussion

We presented a case of a patient with postintubation tracheal stenosis and subsequent acquired laryngomalacia whose shortness of breath was initially attributed to PVFM, which was influenced by the presence of comorbid depression, anxiety, and borderline personality disorder. This case highlights the logical fallacy that may occur in patients with underlying psychiatric disease and an organic illness, in which the patient may present with signs and symptoms that may initially appear as one diagnosis, when the actual underlying diagnosis may be something completely different. This calls attention to the critical importance of a thorough workup in patients who present with upper respiratory distress and psychiatric disease.

The association between psychiatric and physical diseases is well-established, with patients affected by psychiatric diseases exhibiting higher morbidity and mortality rates in the presence of physical ailments [5]. Moreover, the diagnosis, prognosis, and treatment of physical diseases can be complicated by the presence of psychiatric disorders [6]. PVFM, a condition characterized by vocal cord closure during inspiration, leading to wheezing, inspiratory stridor, and shortness of breath, has a known link to psychiatric disease [3,7]. PVFM has been linked to psychiatric disorders since its discovery. It was initially called hysterical croup and has since also been referred to as psychogenic stridor and Munchausen’s stridor [8]. Up to 70% of patients with PVFM present with an associated psychological component or an underlying psychiatric diagnosis [4]. The connection between PVFM and psychiatric disorders is increasingly evident in recent studies. In a notable case series, the coexistence of PVFM and psychiatric conditions was revealed, with four out of five patients diagnosed with PVFM also presenting a concomitant psychiatric disorder [9]. Furthermore, a separate investigation found that among 48 PVFM patients, 45 individuals carried a psychiatric diagnosis, with conversion disorder accounting for more than half of these cases [10]. It is noteworthy, however, that the interplay between psychiatric disorders and PVFM is complex. Some studies suggest that conditions such as depression and anxiety - frequently linked with PVFM - could potentially be consequences of the PVFM symptoms rather than root causes of the condition itself [11-13].

PVFM exhibits a distinct demographic pattern, with the highest prevalence observed in females, young adults, and athletes [14]. This condition presents a bimodal distribution, manifesting in both adults and the pediatric population. Specifically, the median age of onset for adults is approximately 36.5 years, while pediatric patients tend to experience the median onset at 14 years [15]. In adults, PVFM displays a noticeable gender disparity, with a female-to-male ratio exceeding 2:1. Conversely, in the pediatric context, the demographic most commonly affected by PVFM is young, highly competitive female athletes, highlighting the unique presentation and susceptibility of this subgroup [16].

Tracheal stenosis is a recognized complication following prolonged endotracheal intubation, presenting with symptoms that can overlap with PVFM, including wheezing, stridor, and shortness of breath. Although intubation is the leading cause of tracheal stenosis, its incidence postintubation ranges from 1% to 21% [17]. Repeated intubations significantly increase the risk of tracheal stenosis and this patient's history of multiple intubations likely led to her developing stenosis. Tracheal stenosis can develop due to necrosis-induced pressure-related loss of blood flow, leading to mucosal and structural airway damage, ultimately resulting in airway narrowing and respiratory distress [18]. Acquired laryngomalacia is a very rare disease in adults that occurs due to the collapse of supraglottic structures during inspiration and in this patient may be due to the Bernoulli effect causing repeated insults to supraglottic structures and leading to the development of edema and redundant mucosa [19].

The treatment approaches for tracheal stenosis, acquired laryngomalacia, and PVFM all share a common goal: to alleviate the distressing dyspnea experienced by patients. However, the definitive strategies for each condition differ based on symptom severity and individual cases. Among these conditions, tracheal stenosis often requires surgical intervention and can be treated with in-office procedures and operating room endoscopic or open approaches. Acquired laryngomalacia, being a relatively rare and less documented condition, lacks a consensus on treatment protocols. Nevertheless, laser supraglottoplasty is a commonly utilized intervention [2]. Supplementary methods, including laser epiglottidectomy and defunctioning tracheostomy, have also found application [2]. In the case of our patient, extensive treatment for tracheal stenosis and multiple intubations led to the performance of laser supraglottoplasty, aimed at addressing the acquired laryngomalacia.

PVFM treatment primarily revolves around respiratory retraining therapy, guided by a speech-language pathologist [20]. Additionally, acknowledging the often-contributory role of underlying psychiatric issues is crucial in managing the symptoms and severity of PVFM [20]. It is this multifaceted approach that underlies the successful management of PVFM, emphasizing both physical and psychological aspects.

## Conclusions

This case report exemplifies the diagnostic challenges posed by patients presenting with upper respiratory distress who also have comorbid psychiatric disease. Our patient was initially misdiagnosed with PVFM due to exam findings combined with her comorbid psychiatric diagnosis. Further workup showed tracheal stenosis and acquired laryngomalacia as the underlying conditions. The association between physical complaints and psychiatric disorders complicates both the diagnosis and treatment, with psychiatric conditions often worsening morbidity and mortality rates. Treatment for PVFM, tracheal stenosis, and acquired laryngomalacia vary and thus timely and comprehensive evaluations, including a thorough medical and psychiatric history, are crucial in order to treat both medical and psychosomatic conditions.
